# The correlation between peripheral complete blood count parameters and diabetic macular edema in proliferative diabetic retinopathy patients: a cross-sectional study

**DOI:** 10.3389/fendo.2023.1190239

**Published:** 2023-07-19

**Authors:** Chunyan Lei, Jinyue Gu, Lili Liu, Keren Zhang, Meixia Zhang

**Affiliations:** ^1^ Department of Ophthalmology, West China Hospital, Sichuan University, Chengdu, China; ^2^ Research Laboratory of Macular Disease, West China Hospital, Sichuan University, Chengdu, China; ^3^ Department of Ophthalmology, Zigong First People’s Hospital, Zigong, China

**Keywords:** diabetic macular edema, proliferative diabetic retinopathy, peripheral complete blood count, inflammation, white blood cell (WBC)

## Abstract

**Background:**

Numerous studies have demonstrated that retinal chronic inflammation plays a critical role in the pathogenesis of diabetic macular edema (DME). However, studies about the association between peripheral complete blood count, an inexpensive and easily measurable laboratory index, and DME are limited.

**Research design and methods:**

The current study was a hospital-based, cross-sectional study. The participants were inpatients with type 2 diabetes who underwent vitrectomy for PDR, and the contralateral eyes in these PDR patients meeting the criteria were included in the study. Central macular thickness (CMT) was measured automatically and the DME was characterized as CMT ≥ 300 μm.

**Results:**

A total of 239 PDR participants were enrolled. The average age was 55.46 ± 10.08 years old, and the average CMT was 284.23 ± 122.09 μm. In the fully adjusted model, for CMT, the results revealed a significantly negative association between CMT and both white blood cell (WBC) count and neutrophil count (β = −11.95, 95% CI: −22.08, −1.82; *p* = 0.0218; β = −14.96, 95% CI: −28.02, −1.90; *p* = 0.0259, respectively); for DME, the results showed an inverse association between DME and WBC count, monocyte count, and eosinophil count (OR = 0.75, 95% CI: 0.59, 0.95; *p* = 0.0153; OR = 0.07, 95% CI: 0.00, 0.92; *p* = 0.0431; OR = 0.03, 95% CI: 0.00, 0.88; *p* = 0.0420, respectively).

**Conclusions:**

In conclusion, our results suggest that WBC and its subtypes in circulation may play an important role in the pathogenesis of DME in PDR patients.

## Introduction

Among the working-age population in developed countries, diabetic retinopathy (DR) is the leading cause of visual impairment (1). Proliferative DR (PDR) is the most advanced stage of DR and is characterized by neovascularization and proliferative membrane formation, which can cause vitreous hemorrhage and tractional retinal detachment, leading to progressive vision loss (2–5). Diabetic macular edema (DME) is caused by the accumulation of intra-retinal fluid due to the breakdown of blood–retinal barrier, resulting in inflammatory changes and retinal thickening (6). DME as a sight-threatening complication can occur at any stage of DR; the prevalence of DME is related to the duration of diabetes mellitus (DM) duration and the severity of DR. PDR and DME frequently co-occur, with the prevalence of DME ranging from 30% to 72.6% in patients with PDR (7, 8).

Previous studies have shown that DM and its microvascular complications are associated with chronic inflammation (9–11). These findings suggested an interaction between inflammation and the pathogenesis of DR. Substantial evidence indicates that retinal inflammation plays a crucial role in the pathogenesis of DME (12), which is well illustrated by current treatment of DME, including intravitreal injection of anti-vascular endothelial growth factor (VEGF) drugs (13, 14) and anti-inflammatory therapy (Ozurdex, triamcinolone acetonide, etc.) (15, 16). The complete blood count is an affordable and readily available test; white blood cell (WBC, also known as leukocyte) and its subtypes are considered to be the biomarkers of inflammatory response, as their activation results in the synthesis of inflammatory cytokines. Recently, increasing concern has been demanded regarding WBC and its subtypes and its relations in several ocular inflammatory conditions such as uveitis (17–19), DR (20–22), and DME (23–25). All these inflammation indexes derived by peripheral complete blood count parameters including the neutrophil-to-lymphocyte ratio (NLR) (24, 26) and systemic immune-inflammation index (SII, calculated as platelet count*neutrophil count/lymphocyte count) (24, 27) have been investigated as potential biomarkers for predicting or guiding treatment in DME.

The risk factors for DME have been extensively explored, including DM duration (28), hypertension (29, 30), glycosylated hemoglobin (HbA1c) levels (31–33), and other factors (29). Numerous studies have demonstrated that retinal chronic inflammation plays a critical role in the pathogenesis of DME. However, whether WBC in circulation is related to DME in PDR patients is still unknown and there have been limited reports in the literature investigating risk factors for DME in patients with PDR. Therefore, we aimed to employ an inexpensive and effective method to study the potential relationship between peripheral blood biomarkers and DME in PDR patients with type 2 DM by blood cell count or blood cell count-derived index.

## Materials and methods

### Study population

The current study was a hospital-based, cross-sectional study. The study protocol followed the principles of the Declaration of Helsinki and the trial was ethically approved by the West China Hospital of Sichuan University (2020-834). The data were anonymous; therefore, the requirement for informed consent was waived. The participants were inpatients with type 2 diabetes who underwent vitrectomy for PDR at the Ophthalmology Department of West China Hospital of Sichuan University from May 2020 to February 2022, and the contralateral eyes in these PDR patients meeting the criteria were included in the study.

Inclusion criteria were as follows: (1) the contralateral PDR-graded eyes of inpatients undergoing vitrectomy for PDR with clear refractive media and no history of vitrectomy; (2) fasting blood glucose lower than 8 mmol/L; blood glucose < 11 mmol/L 2 h after three meals; consistent blood glucose levels for at least 7 days; and (3) no missing peripheral complete blood count parameters and central macular thickness (CMT). Exclusion criteria were as follows: (1) refractive error exceeding ± 6.00 diopters, or an axial length of more than 26.50 mm; (2) acquired immune deficiency syndrome, syphilis, or leukemia; (3) type 1 diabetes; and (4) neovascular glaucoma or iris neovascularization, retinal vein occlusion, retinal artery occlusion, uveitis, age-related macular degeneration, paracentral acute middle maculopathy, ocular trauma, endophthalmitis, and vitreomacular interface abnormalities, such as vitreomacular traction and epiretinal membrane.

### Laboratory variables

For all relevant laboratory tests, all participants underwent a forearm venous puncture for peripheral overnight fasting blood (fasting for at least 8 h) extraction and were sent to the central laboratory for measurement in 2 h. Complete blood counts were obtained using an automated hematology analyzer (Japan Sysmex company’s automatic XN-9000/XN-9100/XN3100 five-classification blood cell analyzer) for detection. Complete blood count parameters were as follows: red blood count (RBC), hemoglobin, platelet count, and WBC and its subtypes including neutrophil, lymphocyte, monocyte, eosinophil, and basophil. In addition to complete blood counts themselves, we also used indicators generated by parameters in whole blood cells such as NLR, platelet-to-lymphocyte ratio (PLR), lymphocyte-to-monocyte ratio (LMR), and SII; the aforementioned indicators have been investigated as potential risk markers in several clinical conditions. Fasting laboratory values included serum lipid profile, fasting blood glucose, preoperative HbA1c, serum creatinine, and estimated glomerular filtration rate (eGFR).

### Optical coherence tomography imaging

All participants underwent comprehensive ocular examinations on admission (on the same day), including visual acuity, intraocular pressure, axial length, slit-lamp examination, and enhanced depth imaging-optical coherence tomography (OCT). The OCT examination was conducted after pupil dilation with compound tropicamide eye drops (Mydrin-P; Santen, Osaka, Japan). The standardized OCT scans were obtained using spectral-domain (SD) OCT (Heidelberg Engineering; Heidelberg, Germany) by horizontal/vertical scans and carried out by experienced technicians in the afternoon. The CMT centered on the fovea was measured automatically. CMT was defined as the vertical distance from the macular inner limiting membrane to the retinal pigment epithelium. The measurements were performed independently by two experienced physicians blinded to patients’ clinical data, and the average of all measurements was used for the final statistical analysis.

Patients with CMT measured as ≥300 μm could perform intravitreal anti-VEGF inhibitors or steroid injections (34). Therefore, in the current study, the DME was characterized as CMT ≥ 300 μm.

### Other variables

The relevant baseline characteristics that were important in the management of hospitalized patients with PDR were retrieved from the electronic medical record system. Demographic information included age, sex, and educational level. The educational level was grouped into less than 12th grade, high school, and college or above. Hypertension history was defined as physician diagnosis or the use of antihypertensive medications. DM was defined as physician diagnosis or the use of insulin/diabetic tablets. Relevant medical history included the duration of diabetes, hypertension, chronic kidney disease, stroke, and heart disease. The degree of panretinal photocoagulation (PRP) in the contralateral eyes of patients with PDR and the history of anti-VEGF treatment were collected. The degree of PRP was grouped into three categories, none, partial, and whole, according to the scope of the laser. All of the above data were obtained from the medical conditions that were self-reported by participants. Systemic medication history was extracted, including oral glucose-lowering drugs, insulin treatment, and oral antihypertensive drugs. Physical characteristics were extracted for the initial presentation, including height, weight, and systolic and diastolic blood pressure. The body mass index (BMI) was calculated as the weight (kilogram) divided by the height (meter) squared.

### Statistical analysis

Demographic characteristics and findings were summarized using descriptive statistics. Continuous variables were summarized as means ± standard deviation, and the categorical variables were summarized as frequencies and percentages. We compared the data distribution of each covariate between two groups with or without DME using the *t*-test (normal distribution) or Kruskal–Wallis rank-sum test (non-normal distribution) for continuous variables and *χ* ^2^ tests for categorical data. Multivariate linear regression analysis was used to detect the independent association of complete blood count parameters with CMT, and logistic regression analysis was used to detect the independent association of complete blood count parameters with DME.

We selected candidate confounders based on their associations with the outcomes of interest or a change in effect estimate of greater than 10% (35). Statistical analyses were performed using R software, version 3.4.3 (http://www.R-project.org/, The R Foundation) and Empower Stats (http://www.empowerstats.com; X&Y Solutions Inc., Boston, MA). A two-sided *p* < 0.05 was considered to be statistically significant.

## Results

### Baseline characteristics of participants

A total of 239 participants with PDR were enrolled in the final analysis. The average age of the participants was 55.46 ± 10.08 years old, and 58.16% of them were men. The average CMT was 284.23 ± 122.09 μm and the average axial length was 23.18 ± 0.98 mm. Except for BMI, visual acuity (logMAR), WBC count, neutrophil count, monocyte count, direct bilirubin, and anti-VEGF therapy, there was no statistical difference in other variables between the two groups. Baseline characteristics of participants are shown in **Table 1**.

**Table 1:** Baseline characteristics of participants.

	Total	No DME group	DME group	P-value
No.	239	173	66	
Age (year)	55.46 ± 10.08	55.11 ± 9.94	56.36 ± 10.47	0.391
Male sex, n (%)	139 (58.16%)	102 (58.96%)	37 (56.06%)	0.685
Educational level, n (%)				0.585
Less than 12th grade	150 (63.56%)	106 (61.63%)	44 (68.75%)	
High school	41 (17.37%)	31 (18.02%)	10 (15.62%)	
College or above	45 (19.07%)	35 (20.35%)	10 (15.62%)	
BMI (kg/m2)	24.04 ± 3.07	24.32 ± 3.23	23.24 ± 2.43	0.021
SBP (mmHg)	136.66 ± 20.04	137.04 ± 20.71	135.65 ± 18.29	0.647
DBP (mmHg)	82.88 ± 10.76	83.18 ± 10.73	82.10 ± 10.89	0.507
Duration of diabetes (year)	12.23 ± 6.74	12.02 ± 6.64	12.77 ± 7.03	0.443
Ocular parameters				
Visual acuity (logMAR)	0.74 ± 0.54	0.67 ± 0.53	0.95 ± 0.48	<0.001
IOP (mmHg)	16.18 ± 3.87	15.98 ± 3.69	16.70 ± 4.31	0.208
Axial length (mm)	23.18 ± 0.98	23.21 ± 0.97	23.08 ± 1.02	0.539
CMT (μm)	284.23 ± 122.09	220.53 ± 39.64	451.19 ± 106.39	<0.001
Complete blood count				
RBC count, ×10 12/L	4.29 ± 0.65	4.30 ± 0.67	4.24 ± 0.61	0.502
Hemoglobin, g/L	125.64 ± 18.94	126.01 ± 19.72	124.68 ± 16.83	0.629
Platelet count, ×10 10/L	188.52 ± 62.41	188.53 ± 64.48	188.50 ± 57.12	0.998
WBC count, ×10 9/L	6.69 ± 1.64	6.86 ± 1.67	6.25 ± 1.46	0.010
Neutrophil count, ×10 9/L	4.31 ± 1.27	4.44 ± 1.29	4.00 ± 1.14	0.016
Lymphocyte count, ×10 9/L	1.72 ± 0.58	1.75 ± 0.59	1.63 ± 0.57	0.144
Monocyte count, ×10 9/L	0.44 ± 0.15	0.45 ± 0.16	0.40 ± 0.10	0.012
Eosinophil count, ×10 9/L	0.17 ± 0.14	0.17 ± 0.14	0.15 ± 0.13	0.322
Basophil count, ×10 9/L	0.04 ± 0.02	0.04 ± 0.02	0.04 ± 0.02	0.900
Index				
NLR	2.79 ± 1.21	2.81 ± 1.24	2.73 ± 1.15	0.630
LMR	4.20 ± 1.56	4.19 ± 1.59	4.24 ± 1.51	0.839
PLR	118.05 ± 46.14	115.20 ± 45.78	125.48 ± 46.60	0.124
SII	516.60 ± 278.41	519.67 ± 290.43	508.64 ± 246.45	0.785
Other laboratory data				
Total bilirubin (µmol/L)	9.59 ± 4.02	9.79 ± 4.22	9.08 ± 3.43	0.358
Direct bilirubin (µmol/L)	2.54 ± 1.29	2.67 ± 1.38	2.21 ± 0.94	0.043
Indirect bilirubin (µmol/L)	7.04 ± 3.08	7.11 ± 3.15	6.87 ± 2.92	0.650
Fasting blood glucose (mmol/L)	7.26 ± 2.52	7.20 ± 2.37	7.41 ± 2.90	0.557
Serum creatinine (µmol/L)	145.78 ± 180.60	150.29 ± 193.50	133.97 ± 141.96	0.533
eGFR (ml/min×1.73m 2)	66.88 ± 30.36	67.11 ± 30.68	66.28 ± 29.72	0.853
Triglyceride (mmol/L)	1.49 ± 0.75	1.54 ± 0.79	1.37 ± 0.62	0.226
Total cholesterol (mmol/L)	4.58 ± 1.19	4.51 ± 1.13	4.75 ± 1.32	0.282
HDL-C (mmol/L)	1.33 ± 0.47	1.28 ± 0.42	1.45 ± 0.57	0.063
LDL-C (mmol/L)	2.66 ± 1.03	2.62 ± 0.93	2.76 ± 1.24	0.475
HbA1c (%)	7.63 ± 1.60	7.59 ± 1.71	7.72 ± 1.35	0.710
Systemic diseases, n (%)				
Hypertension	113 (47.28%)	83 (47.98%)	30 (45.45%)	0.727
Diabetic nephropathy history	47 (19.67%)	35 (20.23%)	12 (18.18%)	0.722
Stroke history	12 (5.02%)	10 (5.78%)	2 (3.03%)	0.384
Anemia	8 (3.35%)	5 (2.89%)	3 (4.55%)	0.525
Heart disease	12 (5.02%)	10 (5.78%)	2 (3.03%)	0.384
Treatment, n (%)				
PRP degree				0.256
None	114 (47.70%)	88 (50.87%)	26 (39.39%)	
Partial	44 (18.41%)	31 (17.92%)	13 (19.70%)	
Whole	81 (33.89%)	54 (31.21%)	27 (40.91%)	
Anti-VEGF therapy	60 (25.10%)	37 (21.39%)	23 (34.85%)	0.032
Insulin	123 (51.46%)	86 (49.71%)	37 (56.06%)	0.380
Oral glucose-lowering drugs	178 (74.48%)	130 (75.14%)	48 (72.73%)	0.702
Calcium antagonist	50 (21.01%)	37 (21.39%)	13 (20.00%)	0.815

SBP, systolic blood pressure; DBP, diastolic blood pressure; BMI, body mass index; HbA1c, hemoglobin A1c; logMAR, logarithmic minimum resolution angle; IOP, intraocular pressure; HDL-C, high-density lipoprotein cholesterol; LDL-C, low-density lipoprotein cholesterol; eGFR, estimated glomerular filtration rate; CMT, central macular thickness; VEGF, vascular endothelial growth factor; PRP, panretinal photocoagulation; triglyceride; TC, total cholesterol; Hb, hemoglobin; RBC, red blood cell; WBC, white blood cell; NLR, neutrophil-to-lymphocyte ratio; LMR, lymphocyte-to-monocyte ratio; PLR, platelet-to-lymphocyte ratio; SII, systemic immune- inflammation index.

### Univariate analysis

As for CMT, univariate analysis results showed a significant negative correlation between WBC count, neutrophil count, and CMT in participants (β = −12.04, 95% CI: −21.42, −2.67; *p* = 0.0124; β = −17.68, 95% CI: −29.82, −5.54; *p* = 0.0047, respectively). Apart from the factors aforementioned, no other complete blood count parameters were found to be associated with CMT. There was a statistically significant difference in CMT by BMI, visual acuity, and anti-VEGF therapy.

As for DME, univariate analysis results revealed a significant inversely association between WBC count, neutrophil count, monocyte count, and DME in PDR participants (OR = 0.78, 95% CI: 0.64, 0.94; *p* = 0.0108; OR = 0.74, 95% CI: 0.58, 0.95; *p* = 0.0179; OR = 0.06, 0.57, 95% CI: 0.01, 0.57; *p* = 0.0139, respectively). Apart from the factors mentioned above, no other complete blood count parameters were found to be associated with DME. In addition, there was a statistically significant association between BMI, direct bilirubin, visual acuity, anti-VEGF therapy, and DME in PDR participants. The results of univariate analysis are listed in **Table 2**.

**Table 2:** The results of univariate analysis.

		CMT			DME		
		β, 95%CI	P-value		OR, 95%CI	P-value	
Male *vs* female		-2.75 (-34.20, 28.69)	0.8639		0.89 (0.50, 1.57)	0.6847	
Age, per 1 year increase		0.93 (-0.61, 2.46)	0.2384		1.01 (0.98, 1.04)	0.3898	
Educational level							
Less than 12th grade		Ref.			Ref.		
High school		-22.77 (-64.36, 18.82)	0.2844		0.78 (0.35, 1.72)	0.5340	
College or above		-18.86 (-58.97, 21.25)	0.3576		0.69 (0.31, 1.51)	0.3515	
BMI, per 1 kg/m2 increase		-5.47 (-10.46, -0.48)	0.0328		0.89 (0.80, 0.98)	0.0223	
SBP, per 1 mmHg increase		-0.20 (-1.00, 0.60)	0.6224		1.00 (0.98, 1.01)	0.6458	
DBP, per 1 mmHg increase		-0.25 (-1.75, 1.25)	0.7477		0.99 (0.96, 1.02)	0.5052	
Duration of diabetes, per 1 year increase		-0.66 (-2.98, 1.66)	0.5774		1.02 (0.97, 1.06)	0.4416	
Ocular parameters							
Visual acuity, per 1 logMAR increase		47.29 (18.92, 75.67)	0.0012		2.68 (1.57, 4.56)	0.0003	
IOP, per 1 mmHg increase		3.19 (-0.82, 7.20)	0.1207		1.05 (0.97, 1.13)	0.2095	
Axial length, per 1 mm increase		6.41 (-13.29, 26.11)	0.5248		0.87 (0.57, 1.34)	0.5363	
Complete blood count							
RBC count, per 1×1012/L increase		-5.84 (-29.64, 17.96)	0.631		0.86 (0.55, 1.34)	0.5006	
Hemoglobin, per 1 g/L increase		-0.19 (-1.01, 0.63)	0.6481		1.00 (0.98, 1.01)	0.6272	
Platelet count, per 1×10 10/L increase		-0.03 (-0.28, 0.22)	0.7951		1.00 (1.00, 1.00)	0.9977	
WBC count, per 1×10 9/L increase		-12.04 (-21.42, -2.67)	0.0124		0.78 (0.64, 0.94)	0.0108	
Neutrophil count, per 1×10 9/L increase		-17.68 (-29.82, -5.54)	0.0047		0.74 (0.58, 0.95)	0.0179	
Lymphocyte count, per 1×10 9/L increase		-12.15 (-38.84, 14.55)	0.3733		0.68 (0.41, 1.14)	0.1452	
Monocyte count, per 1×10 9/L increase		-78.12 (-182.75, 26.51)	0.1447		0.06 (0.01, 0.57)	0.0139	
Eosinophil count, per 1×10 9/L increase		-12.59 (-126.01, 100.84)	0.828		0.32 (0.03, 3.08)	0.3221	
Basophil count, per 1×10 9/L increase		77.02 (-625.19, 779.23)	0.8300		0.44 (0.00, 174374.31)	0.8997	
Index							
NLR		-7.18 (-20.03, 5.68)	0.2751		0.94 (0.74, 1.20)	0.6285	
LMR		-0.67 (-10.66, 9.33)	0.8961		1.02 (0.85, 1.22)	0.8378	
PLR		0.14 (-0.20, 0.48)	0.4181		1.00 (1.00, 1.01)	0.1253	
SII		-0.03 (-0.08, 0.03)	0.3651		1.00 (1.00, 1.00)	0.7841	
Other laboratory data							
Total bilirubin, per 1µmol/L increase		-1.47 (-5.33, 2.39)	0.4553		0.95 (0.89, 1.03)	0.2269	
Direct bilirubin, per 1µmol/L increase		-10.38 (-22.36, 1.60)	0.0906		0.72 (0.55, 0.93)	0.0137	
Indirect bilirubin, per 1µmol/L increase		-0.66 (-5.70, 4.39)	0.7987		0.98 (0.89, 1.07)	0.6031	
Fasting blood glucose, per 1 mmol/L increase		-0.89 (-7.09, 5.30)	0.7776		1.03 (0.93, 1.16)	0.5554	
Serum creatinine, per 1 µmol/L increase		-0.06 (-0.15, 0.03)	0.1759		1.00 (1.00, 1.00)	0.5345	
eGFR, per 1 ml/min×1.73m 2 increase		-0.09 (-0.60, 0.43)	0.7395		1.00 (0.99, 1.01)	0.8525	
Triglyceride, per 1 mmol/L increase		-5.53 (-34.84, 23.78)	0.7122		0.71 (0.41, 1.24)	0.2268	
Total cholesterol, per 1 mmol/L increase		12.57 (-5.63, 30.78)	0.1781		1.19 (0.87, 1.62)	0.2817	
HDL-C, per 1 mmol/L increase		12.76 (-33.23, 58.74)	0.5875		2.04 (0.91, 4.56)	0.0835	
LDL-C, per 1 mmol/L increase		13.38 (-7.64, 34.40)	0.2145		1.14 (0.80, 1.63)	0.4725
HbA1c, per 1% increase		2.70 (-11.81, 17.21)	0.7161		1.05 (0.81, 1.37)	0.7068
Systemic diseases							
Hypertension *vs* absent		-8.13 (-39.18, 22.92)	0.6082		0.90 (0.51, 1.60)	0.7270
Diabetic nephropathy history *vs* absent		-28.22 (-67.08, 10.64)	0.1560		0.88 (0.42, 1.81)	0.7217
Stroke history *vs* absent		-37.53 (-108.40, 33.34)	0.3003		0.51 (0.11, 2.39)	0.3923
Anemia *vs* absent		17.67 (-68.54, 103.88)	0.6882		1.60 (0.37, 6.89)	0.5282
Heart disease *vs* absent		-31.01 (-101.93, 39.91)	0.3923		0.51 (0.11, 2.39)	0.3923
Treatment							
PRP degree						
None		Ref.			Ref.	
Partial		17.29 (-25.10, 59.68)	0.4248		1.42 (0.65, 3.10)	0.3798
Whole		30.07 (-4.63, 64.78)	0.0908		1.69 (0.90, 3.20)	0.1051
Anti-VEGF therapy *vs* absent		38.14 (2.70, 73.59)	0.0360		1.97 (1.05, 3.67)	0.0335
Insulin *vs* absent		16.57 (-14.39, 47.54)	0.2952		1.29 (0.73, 2.28)	0.3804
Oral glucose-lowering drugs *vs* absent		14.66 (-20.87, 50.19)	0.4194		0.88 (0.46, 1.68)	0.7017
Calcium antagonist *vs* absent		0.07 (-38.14, 38.27)	0.9972		0.92 (0.45, 1.87)	0.8150

SBP, systolic blood pressure; DBP, diastolic blood pressure; BMI, body mass index; HbA1c, hemoglobin A1c; logMAR, logarithmic minimum resolution angle; IOP, intraocular pressure; HDL- C, high-density lipoprotein cholesterol; LDL-C, low-density lipoprotein cholesterol; eGFR, estimated glomerular filtration rate; CMT, central macular thickness; SFCT, subfoveal choroidal thickness; VEGF, vascular endothelial growth factor; PRP, panretinal photocoagulation; triglyceride; TC, total cholesterol; Hb, hemoglobin; RBC, red blood cell; WBC, white blood cell; NLR, neutrophil-to-lymphocyte ratio; LMR, lymphocyte-to-monocyte ratio; PLR, platelet-to- lymphocyte ratio; SII, systemic immune-inflammation index.

### The relationship between complete blood count parameters and both CMT and DME in patients with PDR

We used multivariate linear regression models to assess the relationship between complete blood count parameters and CMT and logistic regression analysis to detect the association between complete blood count parameters and DME in PDR patients (**Table 3**). Three adjusted models are shown in **Table 3**.

**Table 3:** Relationship between complete blood count parameters and central macular thickness/diabetic macular edema in Chinese patients with proliferative diabetic retinopathy in different models.

Exposure(CMT)	Adjusted I model		Adjusted II model		Adjusted III model	
Complete blood count	β, 95%CI	P	β, 95%CI	P	β, 95%CI	P
RBC count, ×10 12/L	-7.00 (-31.19, 17.18)	0.5709	-1.96 (-29.86, 25.93)	0.8904	-5.72 (-33.36, 21.93)	0.6857
Hemoglobin, g/L	-0.27 (-1.11, 0.58)	0.5362	0.21 (-0.78, 1.20)	0.6777	0.17 (-0.81, 1.14)	0.7389
Platelet count, ×10 10/L	-0.03 (-0.28, 0.22)	0.8131	-0.09 (-0.36, 0.18)	0.5156	-0.12 (-0.38, 0.15)	0.4031
WBC, ×10 9/L	-12.06 (-21.50, -2.62)	0.0129	-10.65 (-20.88, -0.43)	0.0424	-11.95 (-22.08, -1.82)	0.0218
Neutrophil count, ×10 9/L	-17.51 (-29.77, -5.24)	0.0056	-13.27 (-26.34, -0.20)	0.0479	-14.96 (-28.02, -1.90)	0.0259
Lymphocyte count, ×10 9/L	-12.92 (-39.75, 13.90)	0.3459	-14.01 (-43.62, 15.60)	0.3548	-17.27 (-46.68, 12.15)	0.2513
Monocyte count, ×10 9/L	-83.92 (-191.21, 23.38)	0.1266	-44.41 (-153.71, 64.88)	0.4267	-57.14 (-165.68, 51.39)	0.3033
Eosinophil count, ×10 9/L	-19.19 (-136.05, 97.68)	0.7479	-116.70 (-249.19, 15.79)	0.0858	-130.25 (-261.14, 0.64)	0.0525
Basophil count, ×10 9/L	86.29 (-627.49, 800.06)	0.8129	66.00 (-700.64, 832.65)	0.8662	60.44 (-701.06, 821.94)	0.8765
NLR	-6.83 (-19.80, 6.14)	0.3030	-2.18 (-15.53, 11.16)	0.7489	-2.46 (-15.77, 10.86)	0.7181
LMR	-0.65 (-11.04, 9.75)	0.9033	-5.57 (-17.44, 6.29)	0.3584	-5.65 (-17.39, 6.09)	0.3469
PLR	0.14 (-0.20, 0.48)	0.4079	0.10 (-0.25, 0.45)	0.5658	0.10 (-0.25, 0.45)	0.5779
SII	-0.02 (-0.08, 0.03)	0.3870	-0.02 (-0.08, 0.04)	0.4447	-0.03 (-0.09, 0.03)	0.3486
Exposure(DME)						
**Complete blood count**	OR, 95%CI	P	OR, 95%CI	P	OR, 95%CI	P
RBC count, ×10 12/L	0.85 (0.54, 1.34)	0.4804	1.06 (0.59, 1.87)	0.8519	0.99 (0.55, 1.80)	0.9798
Hemoglobin, g/L	1.00 (0.98, 1.01)	0.5761	1.01 (0.99, 1.03)	0.5005	1.01 (0.99, 1.03)	0.5102
Platelet count, ×10 10/L	1.00 (1.00, 1.00)	0.9900	1.00 (0.99, 1.00)	0.4604	1.00 (0.99, 1.00)	0.3457
WBC, ×10 9/L	0.78 (0.64, 0.95)	0.0118	0.78 (0.63, 0.98)	0.0325	0.75 (0.59, 0.95)	0.0153
Neutrophil count, ×10 9/L	0.74 (0.58, 0.96)	0.0207	0.80 (0.60, 1.05)	0.1075	0.75 (0.56, 1.01)	0.0557
Lymphocyte count, ×10 9/L	0.67 (0.40, 1.12)	0.1301	0.57 (0.31, 1.07)	0.0781	0.54 (0.29, 1.02)	0.0562
Monocyte count, ×10 9/L	0.05 (0.01, 0.54)	0.0132	0.11 (0.01, 1.34)	0.0839	0.07 (0.00, 0.92)	0.0431
Eosinophil count, ×10 9/L	0.30 (0.03, 3.10)	0.3120	0.06 (0.00, 1.35)	0.0762	0.03 (0.00, 0.88)	0.0420
Basophil count, ×10 9/L	0.65 (0.00, 326897.50)	0.9490	3.09 (0.00, inf.)	0.8866	3.05 (0.00, inf.)	0.8910
NLR	0.95 (0.75, 1.21)	0.6785	1.04 (0.80, 1.36)	0.7749	1.02 (0.78, 1.35)	0.8612
LMR	1.01 (0.84, 1.22)	0.8952	0.88 (0.69, 1.13)	0.3195	0.89 (0.69, 1.14)	0.3389
PLR	1.00 (1.00, 1.01)	0.1246	1.00 (1.00, 1.01)	0.2752	1.00 (1.00, 1.01)	0.3093
SII	1.00 (1.00, 1.00)	0.8143	1.00 (1.00, 1.00)	0.7088	1.00 (1.00, 1.00)	0.5291

Adjust I model adjust for: sex and age.

Adjust II model adjust for: sex, age, duration of diabetes, hypertension, HbA1c, total cholesterol, BMI, eGFR, and direct bilirubin.

Adjust III model adjust for: sex, age, duration of diabetes, hypertension, HbA1c, total cholesterol, BMI, eGFR, direct bilirubin, PRP degree, and anti-VEGF therapy. SBP: systolic blood pressure; DBP, diastolic blood pressure; BMI, body mass index; HbA1c, hemoglobin A1c; logMAR, logarithmic minimum resolution angle; IOP, intraocular pressure; HDL-C, high-density lipoprotein cholesterol; LDL-C, low-density lipoprotein cholesterol; eGFR, estimated glomerular filtration rate; CMT, central macular thickness; SFCT, subfoveal choroidal thickness; VEGF, vascular endothelial growth factor; PRP, panretinal photocoagulation; triglyceride; TC,0 total cholesterol; Hb, hemoglobin; RBC, red blood cell; WBC, white blood cell; NLR, neutrophil-to-lymphocyte ratio; LMR, lymphocyte-to-monocyte ratio; PLR, platelet- to-lymphocyte ratio; SII, systemic immune-inflammation index.

For CMT, in adjusted model I (adjusted age and sex), CMT was significantly associated with WBC count and neutrophil count (β = −12.06, 95% CI: −21.50, −2.62; *p* = 0.0129; β = −17.51, 95% CI: −29.77, −5.24; *p* = 0.0056, respectively). In adjusted model II (adjusted sex, age, duration of diabetes, hypertension, HbA1c, total cholesterol, BMI, eGFR, and direct bilirubin), the results revealed a significantly negative association between CMT and both WBC count and neutrophil count (β = −10.65, 95% CI: −20.88, −0.43; *p* = 0.0424; β = −13.27, 95% CI: −26.34, −0.20; *p* = 0.0479, respectively). Furthermore, in adjusted model III (adjusted sex, age, duration of diabetes, hypertension, HbA1c, total cholesterol, BMI, eGFR, direct bilirubin, PRP degree, and anti-VEGF therapy), the results were consistent (β = −11.95, 95% CI: −22.08, −1.82; *p* = 0.0218; β = −14.96, 95% CI: −28.02, −1.90; *p* = 0.0259, respectively). Apart from the factors aforementioned, no other complete blood count parameters were found to be associated with CMT.

For DME, in adjusted model I (adjusted age and sex), DME was inversely associated with WBC count, neutrophil count, and monocyte count (OR = 0.78, 95% CI: 0.64, 0.95; *p* = 0.0118; OR = 0.74, 95% CI: 0.58, 0.96; *p* = 0.0207; OR = 0.05, 95% CI: 0.01, 0.54; *p* = 0.0132, respectively). In adjusted model II (adjusted sex, age, duration of diabetes, hypertension, HbA1c, total cholesterol, BMI, eGFR, and direct bilirubin), the results revealed a significant association between DME and WBC count (OR = 0.78, 95% CI: 0.63, 0.98; *p* = 0.0325). Furthermore, in adjusted model III (adjusted sex, age, duration of diabetes, hypertension, HbA1c, total cholesterol, BMI, eGFR, direct bilirubin, PRP degree, and anti-VEGF therapy), the results revealed an inverse association between DME and WBC count, monocyte count, eosinophil count (OR = 0.75, 95% CI: 0.59, 0.95; *p* = 0.0153; OR= 0.07, 95% CI: 0.00, 0.92; *p* = 0.0431; OR= 0.03, 95% CI: 0.00, 0.88; *p* = 0.0420, respectively). Apart from the factors aforementioned, no other complete blood count parameters were found to be associated with DME.

### The relationship between inflammation indexes and both CMT and DME in PDR patients

In the present study, all the inflammation indexes derived by peripheral complete blood count parameters including NLR, PLR, LMR, and SII were not associated with CMT/DME. The results are shown in **Table 3**.

## Discussion

The results of the present study provide evidence that lower physiological peripheral WBC levels were associated with increased CMT as well as odds of DME in Chinese patients with PDR. However, WBC subtypes associated with DME as well as CMT are inconsistent, and some indicators are cutoff values. CMT was associated with the neutrophil count, while eosinophil count showed a cutoff value. DME was correlated with monocyte and eosinophil counts, while neutrophil and lymphocyte counts showed a cutoff value. All other laboratory parameters in complete blood count did not reach statistical significance. Taken together, our results indicated that the peripheral WBC concentrations as well as some of its subtypes, even at normal concentrations in the physiological range, might play a crucial role in the pathogenesis of DME in Chinese PDR patients. As far as we know, this is the first study targeting the association between peripheral complete blood count parameters and CMT as well as in patients with PDR.

WBC subtypes include neutrophil, monocyte, lymphocyte, eosinophil, and basophil, and their total numbers make up the WBC count. Neutrophils have previously been implicated in the pathogenesis of DR (20, 36), and their ability to kill microvascular endothelial cells *in vitro* may be related to retinal capillary degeneration observed in diabetes (20, 37). Recently, Lessieur and collaborators (38) confirmed that neutrophil-derived proteases contribute to the pathogenesis of early DR. In short, neutrophils seem to be positively correlated with DR. Few studies have explored the relationship between neutrophils and DME in PDR patients. Contrary to our expectations, our results showed that neutrophils were negatively associated with CMT, while the relationship between neutrophil counts and DME showed a cutoff value.

For peripheral blood monocytes, our results demonstrated that DME was inversely correlated with monocytes. Wan et al. (21) demonstrated that in diabetic adults, decreased peripheral blood monocyte levels are associated with increased odds of DR after adjusting for potential confounders. They suggested that the attraction and influx of monocytes into the retina by adhering to the outer surface of retinal capillaries and disrupting the blood–retinal barrier may reduce the level of monocytes in the peripheral blood (39, 40). For peripheral blood lymphocytes, Zhu et al. (23) found the inverse relationship between and DME, indicating that for patients suffering from severe DR, lymphocyte percentage may be an important diagnostic tool for detecting the onset and progress of DME. Our results showed a critical association between DME and lymphocyte counts, but no association between CMT and lymphocyte counts. Therefore, at present, we are unable to make a firm conclusion about the relationship between DME and lymphocytes, which needs to increase the sample size for further exploration. In the present study, eosinophils were reversely associated with DME and critically related to CMT. While basophils had no relationship with CMT and DME.

Compelling evidence suggests that the elevated physiological WBC count is associated with the presence and severity of DR (41–44) as well as DME (45). A cross-sectional study of 3,776 Chinese diabetic patients by Tong et al. (41) showed that WBC counts within the normal range had a positive correlation with DR. Moradi et al. (42) found that the increase of WBC count in the physiological range was also related to the presence of DR. A study (45) aiming to investigate potential associations between peripheral blood biomarkers and morphological features of retinal imaging in DME patients revealed that the presence of hyperreflective foci on SD-OCT was found to be associated with significantly elevated WBC count. The previous work (46) demonstrated that adherent leukocytes are temporally and spatially associated with retinal endothelial cell damage and death within 1 week of streptozotocin-induced experimental diabetes in rats. Taken together, even within the normal physiological range, leukocytosis appears to be associated with a high incidence of DR and DME. However, the aforementioned results are different from our study. In the current study, we found that lower physiological serum WBC levels were associated with increased CMT as well as the odds of DME in Chinese patients with PDR.

In general, our results suggested that WBC and some subtypes with lower physiological levels have an association with increased CMT as well as increased odds of DME, which is different from other studies. The following reasons might account for this: First, differences in study populations can lead to inconsistent results. The previously studied population included DME patients from all stages of DR. In the present study, all Chinese PDR patients were comparable in their DR stages. Under the unified classification of diabetes and DR staging, it is more reasonable to study the relationship of WBC and its subtypes with CMT and DME. Second, PDR is characterized by neovascularization and proliferative membrane formation. WBCs may enter the retina through the compromised blood–retinal barrier, such as neovascularization. Compared to patients without DME, PDR patients with DME have more severe damage to the blood–retinal barrier, which may lead to an increase in WBCs in the bloodstream entering the eye, leading to a decrease in WBCs and all subtypes in circulation. Last, the sample size or statistical sensitivity to indicators may be responsible for the results between different subtypes and DME as well as CMT.

Increasing evidence has shown that inflammation indexes derived by peripheral complete blood count parameters are associated with DME (24–27). Elbeyli and collaborators (27) revealed that the SII may be a diagnostic biomarker for identifying DME to improve the risk stratification and management of patients with non-PDR. Özata Gündoğdu et al. (24) confirmed that NLR and SII levels were significantly higher in DME with serous macular detachment. There have been limited reports in the literature comparing CMT and DME with LMR and PLR and we have not found any evidence of the association between CMT as well as DME and LMR as well as PLR. In the current study, we failed to find any association between inflammatory indexes and DR as well as DME in Chinese patients with PDR, which is inconsistent with previous studies. We believe that the following were the reasons: First, our study population is PDR patients with type 2 diabetes requiring PPV surgery, which is inconsistent with other studies. Second, the classification of DME is mainly carried out by CMT. Although it reflects some characteristics of DME to a certain extent, it is not classified in detail and accurately. Because the sample of subjects in the previous study was smaller than ours and we could control for almost all possible risk factors for DME, we believe the association between inflammatory indexes and CMT as well as DME has not been established and that inflammatory indexes should not be used as a target for DME monitoring in Chinese PDR patients.

Our study has some advantages. First, we investigated the relationship between not only CMT, but also a dichotomous variable (DME) defined by CMT, and complete blood count, which is useful for assessing the robustness of data analysis. Second, this was a hospital-based cross-sectional study and therefore prone to potential confounding. However, we used strict statistical adjustments to minimize the effect of residual confounders. Last, the results of the present study provide new ideas for studying the relationship between CMT/DME and WBC as well as its subtypes. As biomarkers for the progression of DME in PDR patients, their clinical significance deserves further study.

However, the current study has some limitations. First, the study design was cross-sectional; thus, the causal relationship between CMT as well as DME and WBC as well as its subtypes cannot be determined. These findings should be interpreted with caution, and further longitudinal studies are required. Second, we just used the CMT to define DME, without a more detailed classification. The relationship between both WBC and its subtypes and different types of DME is unclear and needs further study. Moreover, since the study population was all Chinese PDR patients, the generalization of the results may be limited. Despite these limitations, we believe our study is valuable in that we analyzed many clinical and laboratory parameters in PDR subjects and showed a novel clinical risk factor for DME. More well-designed prospective longitudinal studies are necessary to confirm our findings and to further define the role of serum WBC and its subtypes in Chinese patients with PDR.

## Conclusions

In conclusion, this clinical study demonstrated that lower physiological WBC levels are associated with increased CMT as well as the odds of DME in Chinese patients with PDR. Our results suggest that WBC and its subtypes in circulation may play an important role in the pathogenesis of DME in PDR patients.

## Data availability statement

The raw data supporting the conclusions of this article will be made available by the authors, without undue reservation.

## Ethics statement

The study protocol followed the principles of the Declaration of Helsinki and the trial was ethically approved by the West China Hospital of Sichuan University (2020-834). Written informed consent for participation was not required for this study in accordance with the national legislation and the institutional requirements. The data were anonymous and informed consent was waived by the approving Institutional Review Board because of the retrospective nature of the study.

## Author contributions

Conceptualization: CL and MZ. Data collection: JG and KZ. Formal analysis: CL. Validation: JG and LL. Supervision: MZ. Writing—original draft: CL and JG. Writing—review and editing: CL and MZ. All authors contributed to the article and approved the submitted version.
